# Prevalence, Bacterial Load, and Antimicrobial Resistance of *Salmonella* Serovars Isolated From Retail Meat and Meat Products in China

**DOI:** 10.3389/fmicb.2019.02121

**Published:** 2019-09-24

**Authors:** Xiaojuan Yang, Qingping Wu, Jumei Zhang, Jiahui Huang, Ling Chen, Shi Wu, Haiyan Zeng, Juan Wang, Moutong Chen, Haoming Wu, Qihui Gu, Xianhu Wei

**Affiliations:** ^1^College of Food Science, South China Agricultural University, Guangzhou, China; ^2^Guangdong Institute of Microbiology Guangdong Academy of Sciences, State Key Laboratory of Applied Microbiology Southern China, Guangdong Provincial Key Laboratory of Microbial Culture Collection and Application, Guangdong Open Laboratory of Applied Microbiology, Guangzhou, China

**Keywords:** *Salmonella*, serovar, meat, prevalence, enumeration, antimicrobial resistance

## Abstract

*Salmonella* remains the leading cause of reported bacterial foodborne disease in China. Meat products are recognized as one of the major sources of human salmonellosis; however, there is a lack of comprehensive, quantitative data concerning *Salmonella* contamination of these foods. Therefore, the objectives of this study were to investigate the prevalence, bacterial load, and antimicrobial resistance profiles of various *Salmonella* serovars in retail meat across the whole of China. Between July 2011 and June 2016, a total of 807 retail meat samples were collected, covering most provincial capitals in China. Overall, 159 (19.7%) samples tested positive for *Salmonella*. The highest contamination rate occurred in pork (37.3%, *n* = 287), followed by beef (16.1%, *n* = 161), mutton (10.9%, *n* = 92), dumplings (6.6%, *n* = 212), and smoked pork (3.6%, *n* = 55). Most probable number (MPN) analysis revealed that contamination was mainly in the range of 0.3–10 MPN/g among those samples testing positive using this method (*n* = 83), with eight samples exceeding 110 MPN/g. Among the 456 *Salmonella enterica* subsp. *enterica* isolates obtained in this study, 29 serovars and 33 multilocus sequence typing patterns were identified, with *S.* Derby, *S.* Typhimurium, *S.* London, *S.* Rissen, *S.* 1,4,[5],12:i:-, *S.* Weltevreden, and *S.* Enteritidis being the most prevalent. Among the 218 non-duplicate isolates, 181 (83.0%) were resistant to at least one class of antimicrobials and 128 (58.7%) were resistant to at least three classes. High rates of resistance were observed for tetracycline (65.6%), ampicillin (45.4%), trimethoprim-sulfamethoxazole (40.8%), streptomycin (40.4%), and nalidixic acid (35.8%), with the seven most prevalent serovars, except *S.* Weltevreden, showing higher rates of resistance and multidrug resistance compared with the less dominant serovars. Of note, all *S.* Indiana isolates exhibited resistance to extended-spectrum cephalosporins (including ceftriaxone and cefepime), ciprofloxacin, and multiple other classes of antibiotics. Further, two *S*. 1,4,[5],12:i:- isolates showed resistance to imipenem. This study provides systematic and comprehensive data on the prevalence and antimicrobial resistance profiles of various *Salmonella* serovars isolated from meat products in China, indicating their potential risk to public health.

## Introduction

Foodborne diseases caused by *Salmonella* are an important public health concern. Globally, 94 million cases of gastroenteritis and 155,000 deaths are attributed to *Salmonella* each year (Majowicz et al., 2010; Deng et al., 2012). Although more than 2,600 *Salmonella enterica* serovars have been reported (Achtman et al., 2012), the majority of infections are caused by a limited number of serovars, which may vary from country to country and over time (Hendriksen et al., 2011; Van et al., 2012). *S. enterica* subsp. *enterica* serovars Enteritidis and Typhimurium are the most commonly reported serovars associated with human salmonellosis cases worldwide (Hendriksen et al., 2011). *S.* 1,4,[5],12:i:- is a monophasic variant of *S.* Typhimurium and has recently been recognized as a novel serovar and an emerging cause of infection (Yang et al., 2015). Different serovars are associated with different disease potentials (Achtman et al., 2012), making serotype determination particularly important for epidemiological surveillance and disease assessment.

China has a high incidence of salmonellosis (Cui et al., 2009; Deng et al., 2012), with meat products recognized as a significant source of human infection (Yang et al., 2010; Deng et al., 2012). Correspondingly, high rates of *Salmonella* contamination of retail meats such as pork, beef, and mutton have been reported in several cities and provinces in China (Yan et al., 2010; Yang et al., 2010; Li et al., 2014). However, previous surveillance reports from China are based on sampling carried out in only one or a small number of cities, limiting the applicability of the data. As such, there is a lack of comprehensive data concerning *Salmonella* contamination of retail meat products across China as a whole. In particular, studies carrying out direct enumeration of *Salmonella* from retail meat products in China are limited. Thus, the levels of *Salmonella* contamination of meat products and their potential risk to public health have never been evaluated.

In addition, the increasing prevalence of multidrug resistance (MDR) among *Salmonella* isolates is a global concern. Moreover, emerging resistance to extended-spectrum cephalosporins and fluoroquinolones is of extreme importance to public health, as these classes of antibiotics are vital to the management of human cases of salmonellosis (Lunguya et al., 2013). Therefore, in the current study, samples of meat products were examined to provide scientific data for the quantitative assessment of the risks of *Salmonella* to public health. Samples were collected from retail markets in China and assessed to determine the prevalence and contamination rates of *Salmonella*. The resulting *Salmonella* isolates were also characterized to determine their serotypes, genotypes, and antimicrobial resistance profiles.

## Materials and Methods

### Sample Collection

Between July 2011 and June 2016, 807 meat samples were collected, including pork (*n* = 287), beef (*n* = 161), mutton (*n* = 92), dumplings (*n* = 212), and smoked pork (*n* = 55). The sampled meat products were collected from three types of retail stores: supermarkets, fairs, and farmers’ markets, which covered most of the capital cities of the different provinces of China, including Hong Kong and Macao, resulting in a large geographic spread (Supplementary Table S1 and Supplementary Figure S1). Each sample was weighed, labeled, and placed in a separate sterile bag before being immediately transported to the laboratory in an icebox.

### Detection and Enumeration of *Salmonella*

All of the samples were subjected to qualitative and quantitative analysis for *Salmonella*. Qualitative detection was performed as described in National Food Safety Standard GB 4789.4-2010 for the microbiological examination of *Salmonella* (National Food Safety Standards of China). Briefly, 25 g of homogenized samples were added to 225 ml of buffered peptone water (BPW) (Huankai, Guangzhou, China) and incubated overnight at 37°C. Then, 1 ml aliquots of cultures were incubated in 10 ml of selenite cystine broth (SC) (Huankai) at 37°C and 10 ml of tetrathionate brilliant green broth (TTB) at 42°C for 24 h. Loopfuls of SC and TTB cultures were streaked onto xylose-lysine-tergitol 4 (XLT4) selective agar plates (Difco, Detroit, MI, United States) and chromogenic *Salmonella* agar plates (Huankai), then incubated at 37°C for 24 h. Presumptive colonies were picked from each plate, stabbed into a triple sugar iron slant (Huankai), and incubated at 37°C for 24 h. Isolates with typical *Salmonella* phenotypes were further confirmed using API 20E test strips (bioMerieux, Marcy-l’Etoile, France).

The enumeration of *Salmonella* in the samples was determined using the three-tube most probable number (MPN) method. For the MPN method, 25 g of homogenized samples were mixed with 225 ml of BPW (Huankai). Then, 10 ml of this mixture was added to three empty tubes, and transferring in triplicate 1 ml of the mixture into three tubes containing 9 ml of BPW followed by making 10-fold dilution. *Salmonella* detection of each tube was the same with qualitative method. The MPN value was determined on the basis of the number of positive tube(s) in each of the three sets using the MPN table.

### Serotyping and Multilocus Sequence Typing (MLST)

All confirmed *Salmonella* isolates were serotyped by slide agglutination using commercial O and H antisera (Tianrun Bio-Pharmaceutical, Ningbo, China, and S&A Reagents Lab, Bangkok, Thailand) according to the manufacturer’s instructions. The isolates were then further characterized by MLST. MLST was performed using seven housekeeping genes (*aroC*, *dnaN*, *hemD*, *hisD*, *purE*, *sucA*, *thrA*) with the amplification conditions and primers described on the MLST website^1^, while sequence types (ST) were assigned according to the MLST database available from the same site. Cluster analysis was performed using BioNumerics 7.6 software (Applied Maths, Sint-Martens-Latem, Belgium), while a minimum spanning tree generated from the allelic profiles of the isolates was produced.

### Antimicrobial Susceptibility Testing

*Salmonella* isolates were evaluated for antimicrobial resistance using the Kirby–Bauer disk diffusion method according to the Clinical and Laboratory Standards Institute guidelines (Clinical and Laboratory Standards Institute [Clsi], 2018). Susceptibility to the following 22 antibiotics was tested: ampicillin, amoxicillin-clavulanic acid, cefazolin, cefoxitin, ceftriaxone, ceftazidime, cefotaxime, ceftiofur, cefepime, aztreonam, imipenem, gentamicin, kanamycin, amikacin, streptomycin, tetracycline, ciprofloxacin, enrofloxacin, nalidixic acid, trimethoprim-sulfamethoxazole, chloramphenicol, and florfenicol (Oxoid, Basingstoke, United Kingdom).

## Results

### Prevalence and Enumeration of *Salmonella* in Meat Products Collected From Retail Markets

Out of the 807 samples, 159 (19.7%) were positive for *Salmonella.* Pork had the highest prevalence (37.3%, 107/287) of *Salmonella* contamination, followed by beef (16.1%, 26/161), mutton (10.9%, 10/92), dumplings (6.6%, 14/212), and smoked pork (3.6%, 2/55). Of the 83 samples that tested positive using the MPN method, 40 (48.2%) had a contamination level of less than 1 MPN/g, while 25 samples (30.1%) were in the range of 1–10 MPN/g. Ten samples (12.0%) reached 10 MPN/g and eight samples (9.6%) exceeded 110 MPN/g (Table 1).

**TABLE 1:** Prevalence and microbial load of *Salmonella* in retail meat and meat products from China.

**Type of**	**Samples**	**No.(%) Samples**	**No. of samples**			
**products**	**tested no.**	**positive for**				
		***Salmonella***				
			***Salmonella* (MPN/g)**			
			**0.3–1**	**1–10**	**10–110**	**>110**
Pork	287	107 (37.3)	27	22	7	7
Beef	161	26 (16.1)	6	3	3	0
Mutton	92	10 (10.9)	4	0	0	0
Dumpling	212	14 (6.6)	3	0	0	1
Smoked pork	55	2 (3.6)	0	0	0	0
Total	807	159 (19.7)	40	25	10	8

### Serotyping and MLST of *Salmonella*

A total of 456 *Salmonella* isolates were recovered from the 159 positive samples. Based on serotyping and MLST analyses, 29 distinct serovars and 33 STs were identified among the 456 *Salmonella* isolates (Table 2). One isolate belonging to each serotype and ST was selected from each positive sample for further analysis. Thus, 218 non-duplicate isolates were selected from among the 456 *Salmonella* isolates.

**TABLE 2:** Distribution of *Salmonella* serovars and multilocus sequence typing patterns of isolates from retail meat and meat products from China.

**Serotype**	**Total**	**Region of samples**		**Type of samples**					**MLST allelic type**							
		**Southern**	**Northern**					**Smoked**								**MLST pattern**
		**China**	**China**	**Pork**	**Beef**	**Mutton**	**Dumpling**	**pork**	**aroC**	**dnaN**	**hemD**	**hisD**	**purE**	**sucA**	**thrA**	**(no. of isolates)**
*S*. Derby	79	56	19	62	7	3	2	1	19	20	3	20	5	22	22	ST40 (75)
		4	0	3	1	0	0	0	39	35	8	36	29	9	36	ST71 (4)
*S*. Typhimurium	22	13	1	10	0	2	2	0	10	7	12	9	5	9	2	ST19 (14)
		6	2	7	0	0	1	0	10	19	12	9	5	9	2	ST34 (8)
*S*. London	20	16	4	13	4	0	2	1	10	60	58	66	6	65	16	ST155 (20)
*S*. Rissen	18	16	2	15	3	0	0	0	92	107	79	156	64	151	87	ST469 (18)
*S*. 1,4,[5],12:i:-	15	10	5	10	5	0	0	0	10	19	12	9	5	9	2	ST34 (15)
*S*. Weltevreden	13	13	0	10	3	0	0	0	130	97	25	125	84	9	101	ST365 (13)
*S*. Enteritidis	7	4	3	1	1	1	4	0	5	2	3	7	6	6	11	ST11 (7)
S. Meleagridis	4	3	1	4	0	0	0	0	92	125	78	128	138	9	141	ST463 (4)
*S*. Indiana	4	0	4	1	1	0	2	0	8	8	11	11	5	11	15	ST17 (4)
S. Corvallis	3	1	2	1	1	1	0	0	197	187	10	234	8	65	22	ST1541 (3)
*S*. Stanley	3	3	0	2	1	0	0	0	16	16	20	18	8	12	18	ST29 (3)
*S*. Infantis	3	3	0	1	1	0	1	0	17	18	22	17	5	21	19	ST32 (3)
*S*. Kottbus	3	3	0	2	1	0	0	0	14	210	377	14	38	470	12	ST1964 (3)
*S*. Anatum	3	3	0	2	1	0	0	0	10	14	15	31	25	20	33	ST64 (3)
*S*. Thompson	3	0	3	0	1	2	0	0	14	13	18	12	14	18	1	ST26 (3)
*S*. Senftenberg	2	0	1	0	1	0	0	0	7	6	8	8	7	8	13	ST14 (1)
		0	1	0	1	0	0	0	71	65	67	75	61	9	64	ST185 (1)
*S*. Saintpaul	2	1	0	0	1	0	0	0	5	14	18	9	6	12	17	ST27 (1)
		1	0	0	0	1	0	0	5	21	18	9	6	12	17	ST50 (1)
*S*. Wandsworth	2	1	1	0	1	1	0	0	14	13	43	17	96	19	17	ST1498 (2)
*S*. Uganda	2	2	0	1	1	0	0	0	147	13	15	123	15	19	17	ST684 (2)
*S*. Bousso	1	1	0	1	0	0	0	0	222	105	46	123	225	115	115	ST1593 (1)
*S*. Newport	1	1	0	0	1	0	0	0	10	7	21	12	15	12	12	ST46 (1)
*S*. Give	1	0	1	1	0	0	0	0	84	11	16	42	40	71	4	ST516 (1)
*S*. Pomona	1	1	0	1	0	0	0	0	111	109	17	149	41	13	23	ST451 (1)
*S*. Muenster	1	1	0	0	0	1	0	0	119	10	17	42	12	13	4	ST321 (1)
*S*. Mbandaka	1	0	1	0	0	1	0	0	15	70	93	78	113	6	68	ST413 (1)
*S*. Albany	1	1	0	0	1	0	0	0	104	100	54	78	104	9	48	ST292 (1)
*S*. Reading	1	0	1	1	0	0	0	0	46	60	10	9	6	12	17	ST1628 (1)
*S*. Carrau	1	0	1	1	0	0	0	0	84	76	38	16	12	13	4	ST226 (1)
*S*. 4, 5, 12:-:1, 7	1	0	1	0	1	0	0	0	46	430	18	130	8	594	115	ST3134 (1)
**Total**	218	164	54	150	39	13	14	2								

The three most commonly isolated serovars were *S*. Derby (36.2%), *S*. Typhimurium (10.1%), and *S*. London (9.2%), followed by *S*. Rissen (8.3%), *S*. 1,4,[5],12:i:- (6.9%), *S*. Weltevreden (6.0%), and *S*. Enteritidis (3.2%). Notably, two different serovars were simultaneously identified in 31 samples, three different serovars were detected in four samples, four different serovars were detected in four samples, and five different serovars were detected in two samples. *S*. Derby in combination with *S*. Typhimurium was the predominant (18.6%, 8/43) co-contamination pattern.

**FIGURE 1:**
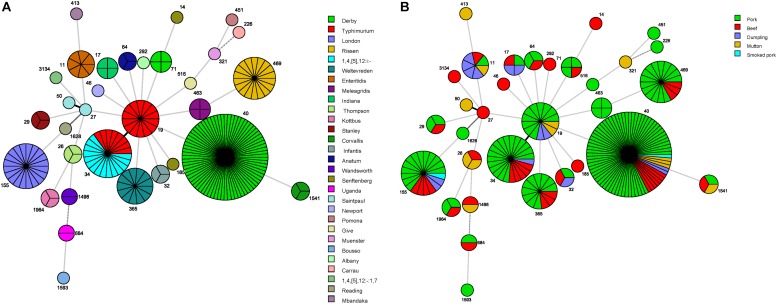
Minimum spanning tree based on multilocus sequence typing data for the 218 *Salmonella* isolates examined in this study. Each circle represents one ST, subdivided into one sector per isolate, flanked by the ST number in small print. The size of circle is related to the number of strains within this ST. The colors in the circles in **(A)** represent the serovars, and the colors in the circles in **(B)** represent the food sources.

A minimum spanning tree based on the concatenated sequences of the seven genes used for MLST analysis revealed the relationships between the 218 *Salmonella* isolates. The STs of the *Salmonella* isolates were then further analyzed relative to serovar and sample type (Figures 1A,B). Among the serovars represented by more than two isolates, only *S*. Derby, *S*. Typhimurium, *S.* Senftenberg, and *S*. Saintpaul showed multiple MLST patterns (Table 2 and Figures 1A,B). Further, only ST34 was associated with multiple *Salmonella* serovars, including eight *S*. Typhimurium isolates and 15 *S*. 1,4,[5],12:i:- isolates.

The serovars were distributed across the different food sources, indicating a high degree of genetic diversity among *Salmonella* strains in China. Overall, the pork meat products contained isolates displaying the greatest ST diversity. Only *S*. Derby was recovered from all five types of meat product (Table 2 and Figures 1A,B) and from all cities sampled in this study (data not shown). Importantly, source-specific differences in the most frequently detected serovars were observed, as follows: pork (*S*. Derby, *S*. Typhimurium, *S*. Rissen); beef (*S*. Derby, *S*. 1,4,[5],12:i:-, *S*. London); mutton (*S*. Derby, *S*. Typhimurium, *S*. Thompson); and dumplings (*S*. Enteritidis, *S*. Typhimurium) (Table 2).

### Antimicrobial Susceptibility Profiles

As shown in Table 3, among the 218 isolates, only 12 (5.5%) were susceptible to all tested antimicrobials. Overall, 181 isolates (83.0%) were resistant to at least one class of antimicrobials and 128 isolates (58.7%) were resistant to at least three classes. A high prevalence of resistance was observed for tetracycline (65.6%), ampicillin (45.4%), trimethoprim-sulfamethoxazole (40.8%), streptomycin (40.4%), and nalidixic acid (35.8%). In addition, 16.1% of isolates showed resistance to ciprofloxacin, with *S*. Derby accounting for the majority of these isolates (20/35), while a high proportion of isolates (34.9%) showed intermediate resistance to this antibiotic. Resistance to third- and fourth-generation cephalosporins ranged from 4.6 to 11.0% among the *Salmonella* isolates. Overall, 5.5% (12/218) and 4.6% (10/218) of the *Salmonella* isolates were resistant to ceftriaxone and cefotaxime, respectively, with *S.* Indiana (four isolates) and *S.* Infantis (two isolates) being the most commonly resistant serovars. Another 11.0% (24/218) of isolates were resistant to ceftazidime, with *S.* Derby (six isolates) and *S.* Enteritidis (five isolates) showing the highest rates of resistance. Of note, 6.4% (14/218) of isolates were resistant to cefepime, with *S.* Indiana (four isolates) and *S.* London, *S.* Enteritidis, *S.* Meleagridis, and *S.* Infantis (two isolates each) representing the most commonly resistant serovars.

**TABLE 3:** Results of antimicrobial susceptibility testing of *Salmonella* isolates obtained in the present study.

**Antimicrobial class**	**Antimicrobial agents**	**No. of isolates (%)**					
		
		**Resistant (R)**		**Intermediate (I)**		**Susceptible (S)**	
Penicillins	Ampicillin (AMP, 10 μg)	99	(45.4)	0	(0.0)	119	(54.6)
β-Lactam/β-lactamase inhibitor combinations	Amoxicillin-clavulanic acid (AMC, 30 μg)	49	(22.5)	27	(12.4)	142	(65.1)
Cephems	Cefazolin (CFZ, 30 μg)	37	(17.0)	49	(22.5)	132	(60.6)
	Cefoxitin (FOX, 30 μg)	16	(7.3)	7	(3.2)	195	(89.4)
	Ceftriaxone (CRO, 30 μg)	12	(5.5)	2	(0.9)	204	(93.6)
	Cefotaxime (CTX, 30 μg)	10	(4.6)	2	(0.9)	206	(94.5)
	Ceftazidime (CAZ, 30 μg)	24	(11.0)	4	(1.8)	190	(87.2)
	Ceftiofur (EFT, 30 μg)	10	(4.6)	8	(3.7)	200	(91.7)
	Cefepime (FEP, 30 μg)	14	(6.4)	9	(4.1)	195	(89.4)
Monobactams Carbapenems	Aztreonam (ATM, 30 μg)	7	(3.2)	3	(1.4)	208	(95.4)
	Imipenem (IPM, 10 μg)	2	(0.9)	6	(2.8)	210	(96.3)
Aminoglycosides	Gentamicin (GEN, 10 μg)	33	(15.1)	11	(5.0)	174	(79.8)
	Kanamycin (KAN, 30 μg)	42	(19.3)	18	(8.3)	158	(72.5)
	Amikacin (AMK, 30 μg)	11	(5.0)	4	(1.8)	203	(93.1)
	Streptomycin (STR, 10 μg)	88	(40.4)	64	(29.4)	66	(30.3)
Tetracyclines	Tetracycline (TET, 30 μg)	143	(65.6)	12	(5.5)	63	(28.9)
Quinolones and fluoroquinolones	Nalidixic acid (NAL, 30 μg)	78	(35.8)	35	(16.1)	105	(48.2)
	Ciproflaxin (CIP, 5 μg)	35	(16.1)	76	(34.9)	107	(49.1)
	Enrofloxacin (ENR, 5 μg)	64	(29.4)	48	(22.0)	106	(48.6)
Folate pathway antagonists	Trimethoprim-sulfamethoxazole (SXT, 25 μg)	89	(40.8)	9	(4.1)	120	(55.0)
Phenicols	Chloramphenicol (CHL, 30 μg)	66	(30.3)	17	(7.8)	135	(61.9)
	Florfenicol (FFC, 30 μg)	76	(34.9)	40	(18.3)	102	(46.8)
	Pansusceptible	12	(5.5)				
	≥ 1 Antimicrobial class	181	(83.0)	≥ 1 Antimicrobial		181	(83.0)
	≥ 3 Antimicrobial class	128	(58.7)	≥ 3 Antimicrobials		132	(60.6)
	≥ 5 Antimicrobial class	78	(35.8)	≥ 6 Antimicrobials		72	(33.0)
	≥ 7 Antimicrobial class	25	(11.5)	≥ 10 Antimicrobials		28	(12.8)
	≥ 9 Antimicrobial class	4	(1.8)	≥ 15 Antimicrobials		6	(2.8)

In addition, all four *S*. Indiana isolates and one *S.* Thompson isolate were identified as being co-resistant to ceftriaxone and ciprofloxacin. These isolates were also resistant to multiple other antimicrobial agents. Of particular concern, three *S*. Indiana isolates showed resistance to all classes of antibiotics tested in the current study, except imipenem. Only two isolates were resistant to imipenem, both of which were *S*. 1,4,[5],12:i:-.

Of the *Salmonella* serovars identified in the present study, *S*. Derby, *S*. Typhimurium, *S*. London, *S*. Rissen, *S*. 1,4,[5],12:i:-, and *S*. Enteritidis had the highest rates of antimicrobial resistance and MDR, while the *S*. Weltevreden isolates were generally quite susceptible to antibiotics (Table 4).

**TABLE 4:** Resistance profiles of the top 15 *Salmonella* serotypes isolated from retail meat and meat products from China.

**Serovars**	**Antimicrobial agents^a^**																						**MDR**				
		
	**AMP**	**AMC**	**CFZ**	**FOX**	**CRO**	**CTX**	**CAZ**	**EFT**	**FEP**	**ATM**	**IPM**	**GEN**	**KAN**	**AMK**	**STR**	**TET**	**CIP**	**ENR**	**NAL**	**SXT**	**CHL**	**FFC**	**≥ 1**	**≥3**	**≥5**	**≥ 7**	**≥9**
Derby (*n* = 79)	33	16 (12)^b^	6 (21)	4 (2)	1 (2)	0	6	0 (2)	1 (1)	0	0 (2)	19 (1)	20 (3)	2 (3)	36 (23)	61 (9)	20 (18)	42 (12)	32 (16)	34 (5)	28 (13)	42 (15)	74	52	32	9	0
Typhimurium (*n* = 22)	16	4 (4)	3 (7)	1 (1)	1	1	1 (1)	1 (1)	0 (2)	0 (1)	0	2 (5)	8 (2)	1 (1)	8 (9)	14 (1)	1 (20)	5 (14)	18	11 (1)	11 (1)	10 (3)	21	16	10	3	0
London (*n* = 20)	10	5 (2)	4 (5)	1 (1)	1	1	2	1	2 (1)	0 (1)	0	5	1 (7)	0	9 (5)	10	0 (11)	5 (4)	0 (10)	10	7 (1)	8 (3)	12	10	8	3	0
Rissen (*n* = 18)	10	5 (2)	3 (7)	0	0	0 (1)	1	0 (1)	0	0	0 (3)	1	1	0	4 (7)	14	1	1 (2)	1	14	1	1 (7)	14	12	4	0	0
1,4,[5],12:i:- (*n* = 15)	14	5 (5)	2 (7)	0	1	0	0	0 (2)	1	0	2	3 (2)	4 (3)	0	11 (2)	14	4 (3)	3 (1)	6 (2)	4	5	5	15	14	8	1	0
Weltevreden (*n* = 13)	0	0	0 (1)	0	0	0	0	0 (1)	0	0	0 (1)	0	0	1	6 (1)	6	0	0 (2)	0 (1)	0	0	0 (2)	7	0	0	0	0
Enteritidis (*n* = 7)	7	5	7	4 (1)	0	0	5	0	2 (3)	0	0	1	1	1	5	2 (2)	1 (5)	0 (3)	7	2 (1)	1	0 (1)	7	7	5	1	0
Meleagridis (*n* = 4)	1	1	1	0	1	1	1	1	2	1	0	0	0	1	1 (1)	4	0 (4)	0 (2)	0 (1)	4	1	1 (1)	4	2	1	1	0
Indiana (*n* = 4)	4	3 (1)	4	2	4	4	2 (1)	4	4	4	0	2 (1)	4	3	2 (1)	4	4	4	4	3 (1)	4	4	4	4	4	4	3
Corvallis (*n* = 3)	0	0	0	0	0	0	0	0	0	0	0	0	0	0	3	3	0 (3)	1	0 (3)	0	0	0 (1)	3	1	0	0	0
Stanley (*n* = 3)	0	0 (1)	1	1 (1)	0	0	1	0	0 (1)	0	0	0 (1)	0	0	1 (1)	1	1	0 (1)	0	0	1	0 (1)	3	1	0	0	0
Infantis (*n* = 3)	2	2	2	2	2	2	2	2	2	2	0	0	1	1	1 (2)	1	0 (3)	0 (2)	3	1	1	0	3	2	2	1	1
Kottbus (*n* = 3)	0	0	0	0	0	0 (1)	0	0	0	0	0	0	0	0	0 (2)	0	0	0	0	0	0	0	0	0	0	0	0
Anatum (*n* = 3)	0	1	1	1	0	0	2	0	0 (1)	0	0	0	1	0	0 (2)	3	0 (2)	0 (3)	1	2	2 (1)	1 (2)	3	3	1	0	0
Thompson (*n* = 3)	1	1	1	0 (1)	1	1	1	1	0	0 (1)	0	0	0	0	1	1	1 (1)	1 (1)	1 (1)	1	1 (1)	1 (1)	2	1	1	1	0

*^*a*^Antimicrobial abbreviations: ampicillin (AMP), amoxicillin-clavulanic acid (AMC), cefazolin (CFZ), cefoxitin (FOX), ceftriaxone (CRO), cefotaxime (CTX), ceftazidime (CAZ), ceftiofur (EFT), cefepime (FEP), aztreonam (ATM), imipenem (IPM), gentamicin (GEN), kanamycin (KAN), amikacin (AMK), streptomycin (STR), tetracycline (TET), ciprofloxacin (CIP), enrofloxacin (ENR), nalidixic acid (NAL), trimethoprim-sulfamethoxazole (SXT), chloramphenicol (CHL), and florfenicol (FFC). ^*b*^The number with parentheses means intermediate resistance.*

## Discussion

### Prevalence of *Salmonella*

This study showed that the overall prevalence rate of *Salmonella* was 19.7% (159/807) for meat products. The isolation rates observed in the current study are similar to those of a previous study in Shaanxi Province, China (Yang et al., 2010). However, the current rates are higher than those obtained in Jiangsu Province, eastern China, where only 14.1% (154/1096) of pork samples were *Salmonella*-positive (Li et al., 2014). Interestingly, a study from Hebei Province, China, reported much higher rates of *Salmonella* contamination of beef and mutton (33.3% for each) compared with pork (26.7%) (Yan et al., 2010). These disparities are likely the result of the different geographic locations of the sampling sites. However, in the current study, samples were collected from 39 cities across China, including most provincial capitals as well as Hong Kong and Macao. To our knowledge, this was the most comprehensive countrywide study of *Salmonella* isolates recovered from meat products in China. Therefore, the resulting data is more comprehensive and representative of China as a whole and will be hugely beneficial for future risk assessment.

Compared with studies conducted in other countries, the prevalence of *Salmonella* contamination of meat products in the current study was lower than the 82% recorded for beef and 93% for pork in Laos (Boonmar et al., 2013). However, the current rates are much higher than those recorded in Canada, where *Salmonella* prevalence rates of only 2.0% and 0% were determined for pork and beef, respectively (Aslam et al., 2012).

### *Salmonella* Serotypes

S. Derby and *S*. Typhimurium were the most prevalent serovars identified in the current study, accounting for 46.3% of all strains. Both serovars were mostly isolated from pork. The prevalence of different *Salmonella* serovars in meat products has been investigated in many areas of China. *S*. Derby followed by *S*. Typhimurium were the two most prevalent serovars among *Salmonella* isolated from retail pork, beef, and lamb samples in Shaanxi Province (Yang et al., 2010) and from pork in Henan (Yang et al., 2013) and Jiangsu (Li et al., 2014) Provinces. While all of these studies focused on a single province, the results were similar to our nationwide data, suggesting that *S*. Derby, followed by *S*. Typhimurium, may be the predominant serovars in livestock-derived meat products across China.

In contrast, studies conducted in different countries have found that other serovars are more common in livestock-derived meat products. A study by Thai et al. (2012) showed that *S*. Anatum was the most common serovar recovered from pork in Vietnam, while in Portugal, *S*. I 4,[5],12:i:- was the predominant serovar in food products of swine and bovine origin (Clemente et al., 2013). However, a high prevalence of *S*. Derby and *S*. Typhimurium was also detected in these two studies (Thai et al., 2012; Clemente et al., 2013). According to a report by Ma̧ka et al. (2014), *S*. Enteritidis was the predominant serotype isolated from pork and beef in Poland, and although *S*. Typhimurium was also isolated, no *S*. Derby isolates were detected. Overall, *S*. Derby and *S*. Typhimurium are reportedly the most common serovars associated with human infection worldwide (Cui et al., 2009; Greig and Ravel, 2009; Deng et al., 2012). Therefore, the high prevalence of these serovars in the current study indicates a significant risk to consumers.

Other serovars that were repeatedly recovered in the present study included *S*. London, *S*. Rissen, *S*. 1,4,[5],12:i:-, *S*. Weltevreden, and *S*. Enteritidis. To the best of our knowledge, *S*. London, *S*. Rissen, and *S*. Weltevreden have rarely been reported in meat by previous studies in China (Yang et al., 2010, 2013; Li et al., 2013, 2014). This suggests that these serovars may be becoming more frequent contaminants of food products in China and should therefore be considered a public health concern.

*S*. 1,4,[5],12:i:-, lacking the phase 2 flagellar antigen has recently been recognized as a monophasic variant of *S.* Typhimurium. Although rarely identified prior to the mid-1990s, the number of human salmonellosis cases caused by *S*. 1,4,[5],12:i:- has increased rapidly in recent years (Yang et al., 2015). Since 2009, *S*. 1,4,[5],12:i:- has also ranked among the four most frequently identified serovars causing human salmonellosis in China (Deng et al., 2012). The prevalence of *S*. 1,4,[5],12:i:- in the current study reminds us that it is critical to monitor the emergence and prevalence of different *Salmonella* serotypes to better control salmonellosis.

*S.* Enteritidis is frequently identified worldwide and is one of the most common serovars associated with human salmonellosis (Greig and Ravel, 2009; Hendriksen et al., 2011). This serovar also accounted for 3.2% of the isolates recovered from meat products in the current study. Of note, *S*. Enteritidis was the predominant serovar isolated from dumplings, indicating poor hygiene practices during dumpling preparation.

Worryingly, most of the *Salmonella* serovars identified in the present study are recognized as frequent causes of human salmonellosis in China (Cui et al., 2009; Deng et al., 2012). Thus, the dissemination of these serovars amongst meat products in China is the likely source of human infections.

### Antimicrobial Susceptibility

Among the isolates recovered in this study, rates of resistance to the various classes of antimicrobial agents ranged from 0.9–65.6%, with resistance to traditional antimicrobial agents such as tetracycline, ampicillin, trimethoprim-sulfamethoxazole, streptomycin, and nalidixic acid being most frequently observed. These results agreed with previous reports from China (Yang et al., 2013; Li et al., 2014), Thailand (Wannaprasat et al., 2011), and Vietnam (Thai et al., 2012). In comparison, rates of resistance among *Salmonella* isolates from meat and dairy products in Egypt were significantly higher, with 95.7 and 91.5% of isolates showing resistance to ampicillin and streptomycin, respectively (Ahmed et al., 2014). In the present study, the highest rates of antimicrobial resistance were recorded for tetracycline (65.6%), which is one of the most widely used antimicrobials in feed additives in livestock farming in China and other countries. The high prevalence of antimicrobial resistance observed here shows the detrimental impact of the uncontrolled use of these antimicrobials for prophylaxis and growth promotion, as well as in medicine, in China.

Multidrug resistance is defined as resistance to at least three classes of antimicrobial agents (Ahmed et al., 2014; Michael and Schwarz, 2016). In total, 128 (58.7%) *Salmonella* isolates showing a MDR phenotype were detected in the current study, a much higher frequency than has been reported by other studies carried out in China (Yang et al., 2013; Li et al., 2014). These results highlight the enormous challenges associated with the treatment of *Salmonella* infections in humans and animals, and further legislation regarding the prudent use of antimicrobials should be implemented by the authorities in China.

As critically important antibiotics in human medicine, extended-spectrum cephalosporins (e.g., ceftriaxone) are the drugs of choice to treat very young patients, while fluoroquinolones (e.g., ciprofloxacin) have been recommended for the treatment of *Salmonella* infections in adults or in cases caused by strains showing extended-spectrum cephalosporin resistance. Resistance to these antibiotics was observed in the current study. Of particular concern was the detection of ciprofloxacin and ceftriaxone co-resistance in all four of the *S.* Indiana isolates and one *S.* Thompson isolate, which poses a significant risk to public health. More seriously, three of the *S*. Indiana isolates showed resistance to all classes of antibiotics tested, except imipenem. Multidrug-resistant *S*. Indiana isolates have been detected previously in China, with most isolated from food-producing animals and humans (Lai et al., 2013; Bai et al., 2015, 2016; Gong et al., 2016). However, to our knowledge, this is the first report of *S*. Indiana isolated from retail meat products exhibiting resistance to extended-spectrum cephalosporins (including ceftriaxone and cefepime), ciprofloxacin, and multiple other antimicrobials in China. These strains are of significant clinical concern because they are unlikely to be adequately controlled by commonly used antibiotics.

Carbapenems (e.g., imipenem) are advocated for the treatment of infections caused by extended-spectrum β-lactamase- and/or AmpC β-lactamase-producing *Enterobacteriaceae*. Unfortunately, the emergence of resistance to carbapenems among *Enterobacteriaceae* has become a global concern. However, the prevalence of imipenem resistance among foodborne *Salmonella* strains in China has not been evaluated (Yan et al., 2010; Yang et al., 2010; Li et al., 2014). Although only two *S*. 1,4,[5],12:i:- isolates showed imipenem resistance in the current study, the dissemination of these strains is a very real threat to public health.

## Conclusion

In summary, *Salmonella* contamination was common in retail meat products in China. In addition to *S*. Typhimurium and *S*. Enteritidis, the two most prevalent *Salmonella* serovars worldwide, numerous other serovars associated with human salmonellosis were identified in the food samples. Moreover, serovar-specific analysis showed that among the dominant serovars, *S*. Derby, *S*. Typhimurium, *S*. London, *S*. Rissen, *S*. 1,4,[5],12:i:-, and *S*. Enteritidis had much higher rates of antimicrobial resistance and MDR, whereas *S*. Weltevreden was generally quite susceptible to all antimicrobial agents. Of note, *S*. Indiana isolates were characterized by their resistance to several extended-spectrum cephalosporins (including ceftriaxone and cefepime), ciprofloxacin, and multiple other antimicrobials, while two *S*. 1,4,[5],12:i:- isolates showed resistance to imipenem. These findings may lead to a greater understanding of the prevalence, load, serotype distribution, genetic diversity, and antimicrobial resistance of *Salmonella* in retail meat products in China. Such data provides support for the development of new approaches to control *Salmonella* infection and antimicrobial resistance.

## Data Availability

All datasets generated for this study are included in the manuscript/Supplementary Files.

## Author Contributions

XY, QW, and JZ conceived and designed the experiments. XY, JH, and LC performed the experiments. XY, SW, HZ, JW, and JZ analyzed the data. MC, HW, QG, and XW contributed reagents, materials, and analysis tools.

## Conflict of Interest Statement

The authors declare that the research was conducted in the absence of any commercial or financial relationships that could be construed as a potential conflict of interest.

## Footnotes

1 http://mlst.warwick.ac.uk/mlst

## Supplementary Material

FIGURE S1 Map of China showing the sampling locations (provinces and cities) of the current study.

TABLE S1 The sampling sites and time of the current study.
