# Supplementary figures and images for: Prevalence, Bacterial Load, and Antimicrobial Resistance of Salmonella Serovars Isolated From Retail Meat and Meat Products in China
Source: Front Microbiol. 2019 Sep 24;10:2121. doi: 10.3389/fmicb.2019.02121 (PMC6771270; doi:10.3389/fmicb.2019.02121)

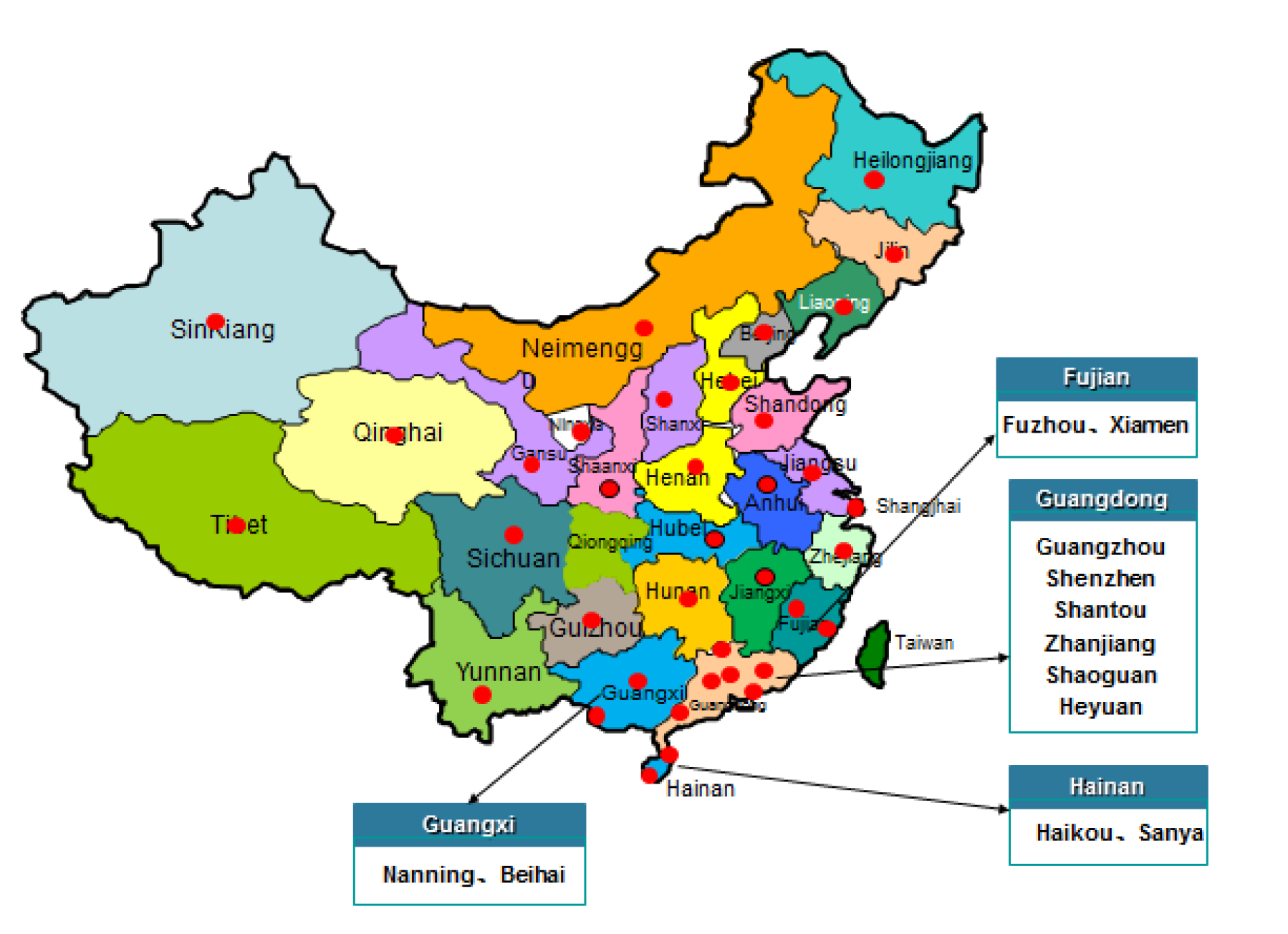

Supplement: FIGURE S1 — Map of China showing the sampling locations (provinces and cities) of the current study. [file Image_1.TIF]
