# Supplementary material for: Prevalence, Bacterial Load, and Antimicrobial Resistance of Salmonella Serovars Isolated From Retail Meat and Meat Products in China
Source: Front Microbiol. 2019 Sep 24;10:2121. doi: 10.3389/fmicb.2019.02121 (PMC6771270; doi:10.3389/fmicb.2019.02121)
Supplement: TABLE S1 — The sampling sites and time of the current study. [file Table_1.docx]

Table S1 The sampling sites and time of the current study

| Sampling site | | Sampling time (year.month) |  |
| --- | --- | --- | --- |
| City | Province |  |  |
| **Southern China** | | |  |
| Guangzhou | Guangdong | 2011.07/2013.09 |  |
| Shenzhen | Guangdong | 2011.12/2013.05 |  |
| Shaoguan | Guangdong | 2012.03/2013.05 |  |
| Zhanjiang | Guangdong | 2012.01/2013.06 |  |
| Shantou | Guangdong | 2012.02/2013.05 |  |
| Heyuan | Guangdong | 2012.03/2013.06 |  |
| Haikou | Hainan | 2012.05/2013.12 |  |
| Sanya | Hainan | 2012.05/2014.01 |  |
| Beihai | Guangxi | 2012.06/2013.12 |  |
| Nanning | Guangxi | 2012.07/2013.11 |  |
| Fuzhou | Fujian | 2012.07/2013.11 |  |
| Xiamen | Fujian | 2012.08/2013.12 |  |
| Macao* | -- | 2015.12/2016.05 |  |
| Hongkong* | -- | 2015.12/2016.05 |  |
| Shanghai | -- | 2012.09/2014.04 |  |
| Hefei | Anhui | 2012.09/2014.03 |  |
| Nanchang | Jiangxi | 2012.09/2014.03 |  |
| Wuhan | Hubei | 2012.10/2014.04 |  |
| Chengdu | Sichuan | 2012.10/2014.03 |  |
| Kunming | Yunnan | 2012.11/2014.05 |  |
| Changsha | Hunan | 2015.06/2016.01 |  |
| Hangzhou | Zhengjiang | 2015.07/2016.02 |  |
| Guiyang | Guizhou | 2015.07/2016.01 |  |
| Nanjing | Jiangsu | 2015.09/2016.03 |  |
| **Northern China** | | |  |
| Lanzhou | Gansu | 2012.11/2013.08 |  |
| Haerbin | Heilongjiang | 2012.11/2013.07 |  |
| Xi’an | Shaanxi | 2012.12/2013.07 |  |
| Taiyuan | Shanxi | 2012.12/2013.08 |  |
| Beijing | -- | 2012.12/2013.08 |  |
| Jinan | Shandong | 2012.12/2013.08 |  |
| Changchun | Jilin | 2015.08/2016.05 |  |
| Xining | Qinghai | 2015.08/2016.05 |  |
| Yinchuan | Ningxia | 2015.08/2016.04 |  |
| Huhehaote | Neimenggu | 2015.08/2016.05 |  |
| Shenyang | Liaoning | 2015.09/2016.03 |  |
| Shijiazhuang | Hebei | 2015.10/2016.03 |  |
| Zhengzhou | Henan | 2015.10/2016.04 |  |
| Lasa | Tibet | 2015.11/2016.05 |  |
| Wulumuqi | Xinjiang | 2015.11/2016.06 |  |

* These two cities were direct-controlled municipalities
